# “CLASSIC NMR”: An In-Situ NMR Strategy for Mapping the Time-Evolution of Crystallization Processes by Combined Liquid-State and Solid-State Measurements**

**DOI:** 10.1002/anie.201404266

**Published:** 2014-07-07

**Authors:** Colan E Hughes, P Andrew Williams, Kenneth D M Harris

**Affiliations:** School of Chemistry, Cardiff UniversityCardiff CF10 3AT, Wales (UK)

**Keywords:** crystal growth, in-situ studies, solid-state nmr spectroscopy, time-dependent processes

## Abstract

A new in-situ NMR strategy (termed CLASSIC NMR) for mapping the evolution of crystallization processes is reported, involving simultaneous measurement of both liquid-state and solid-state NMR spectra as a function of time. This combined strategy allows complementary information to be obtained on the evolution of both the solid and liquid phases during the crystallization process. In particular, as crystallization proceeds (monitored by solid-state NMR), the solution state becomes more dilute, leading to changes in solution-state speciation and the modes of molecular aggregation in solution, which are monitored by liquid-state NMR. The CLASSIC NMR experiment is applied here to yield new insights into the crystallization of m-aminobenzoic acid.

The development of in-situ techniques^1^ to explore crystallization processes from solution promises to yield significant new insights on fundamental aspects of such processes.^2^ With this motivation, we recently developed a new in-situ solid-state NMR technique^3^ which exploits the ability of NMR to selectively observe the solid phase in heterogeneous solid/liquid systems, of the type that exist during crystallization from solution, under conditions in which the liquid phase is “invisible” to the measurement. As a consequence, the technique allows the first solid particles formed during crystallization to be identified, and allows the evolution of different solid phases (e.g., polymorphs^4^) present during the crystallization process to be monitored as a function of time. This in-situ NMR technique has been shown to be a powerful strategy for establishing the sequence of solid phases produced during crystallization^3^ (inter alia, indicating the time-window during which each transient phase is present) and for the discovery of new polymorphs.3c

Here, we report a new development of this in-situ NMR technique that has the potential to yield significantly deeper insights into crystallization processes than the version implemented hitherto. Specifically, the new development exploits the fact that NMR spectroscopy is able to study both the liquid phase *and* the solid phase in a heterogeneous solid/liquid system using the same instrument, simply by changing the pulse sequence used to record the data. Thus, by alternating between two different pulse sequences in an in-situ NMR study of crystallization, alternate solid-state NMR and liquid-state NMR spectra are recorded, yielding essentially simultaneous information on the time-evolution of *both* the solid phase *and* the liquid phase (Figure 1). We call this strategy CLASSIC NMR (Combined Liquid- And Solid-State In-situ Crystallization NMR). We emphasize that the CLASSIC NMR experiment can be carried out on any standard solid-state NMR spectrometer, without requiring modification of the instrumentation.

**Figure 1:**
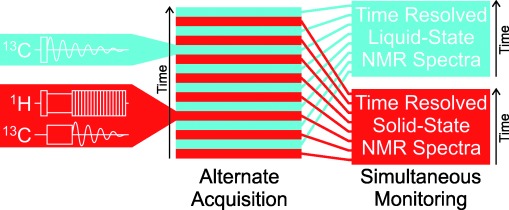
Schematic of the CLASSIC NMR experiment.

Here we demonstrate the advantages of the CLASSIC NMR strategy by applying it to study crystallization of *m*-aminobenzoic acid (*m*-ABA), a system of interest in the context of polymorphism research.^5^ To date, five polymorphs of *m*-ABA have been reported.5a,b The crystal structures of four polymorphs (Forms II, III, IV, and V) are known, while the crystal structure of Form I has not yet been determined. In Forms I, III and IV, the *m*-ABA molecules exist as zwitterions. In Forms II and V, the *m*-ABA molecules are non-zwitterionic. In solution, the tautomeric form of *m*-ABA is solvent dependent,^6^ with *m*-ABA reported to be predominantly zwitterionic in water (proportion of zwitterions ranging from 50 % to 78 %),6a,b almost entirely non-zwitterionic in methanol (proportion of non-zwitterions greater than 98 %)6a and non-zwitterionic in DMSO, 1,4-dioxane and chloroform.6c–e

The aim of the CLASSIC NMR experiment is to elucidate the complementary changes that occur in the solid and liquid phases as crystallization proceeds. The evolution of the crystallization process in terms of both the amount and the polymorphic identity of the solid phase present is established as a function of time from the solid-state NMR spectra. Concomitantly, the solution becomes more dilute as crystallization proceeds, with the changes in solution-state speciation and modes of molecular aggregation in solution monitored from the time-evolution of the liquid-state NMR spectrum. For *m*-ABA, an issue of additional interest concerns the tautomeric forms that exist in the solid and liquid phases.

In the CLASSIC NMR experiment, spectra are recorded using an alternating cycle of two pulse sequences (Figure 1). The key requirement is that one pulse sequence is selective for detecting a signal from the solid phase (ideally with the liquid phase “invisible” to the measurement) and the other pulse sequence is selective for detecting a signal from the liquid phase (ideally with the solid phase “invisible” to the measurement). Clearly, the details of the specific pulse sequences chosen for the solid- and liquid-state measurements may depend on the specific system under investigation. In the present work, ^1^H→^13^C cross-polarization (CP) with high-power ^1^H decoupling has been used to record the high-resolution solid-state ^13^C NMR spectrum, recognizing that, under normal circumstances and for typical values of CP contact time, a signal is observed only from the solid phase. To record the liquid-state ^13^C NMR spectrum, we have used a direct-excitation ^13^C NMR pulse sequence with no ^1^H decoupling and with a relatively short recycle delay (of the magnitude typically used to record liquid-state NMR spectra). The absence of ^1^H decoupling and the short recycle delay ensure that no significant signal is detected from the solid phase.

Initially, high-resolution solid-state ^13^C NMR spectra (Figure 2) were recorded for powder samples of the five polymorphs of *m*-ABA. Clearly, each polymorph is uniquely distinguished by its ^13^C NMR spectrum, which is critical for enabling the polymorph(s) present in subsequent in-situ crystallization experiments to be identified. The ^1^H spin-lattice relaxation times (*T*_1_) for each polymorph at 303 K are: 0.6 s (Form I), 190 s (II), 1.7 s (III), 0.5 s (IV) and 7.2 s (V). Significantly, Forms II and V have much slower spin-lattice relaxation than the other three polymorphs, suggesting that long *T*_1_(^1^H) is associated with non-zwitterionic *m*-ABA molecules in the crystal structure.5b, 7

**Figure 2:**
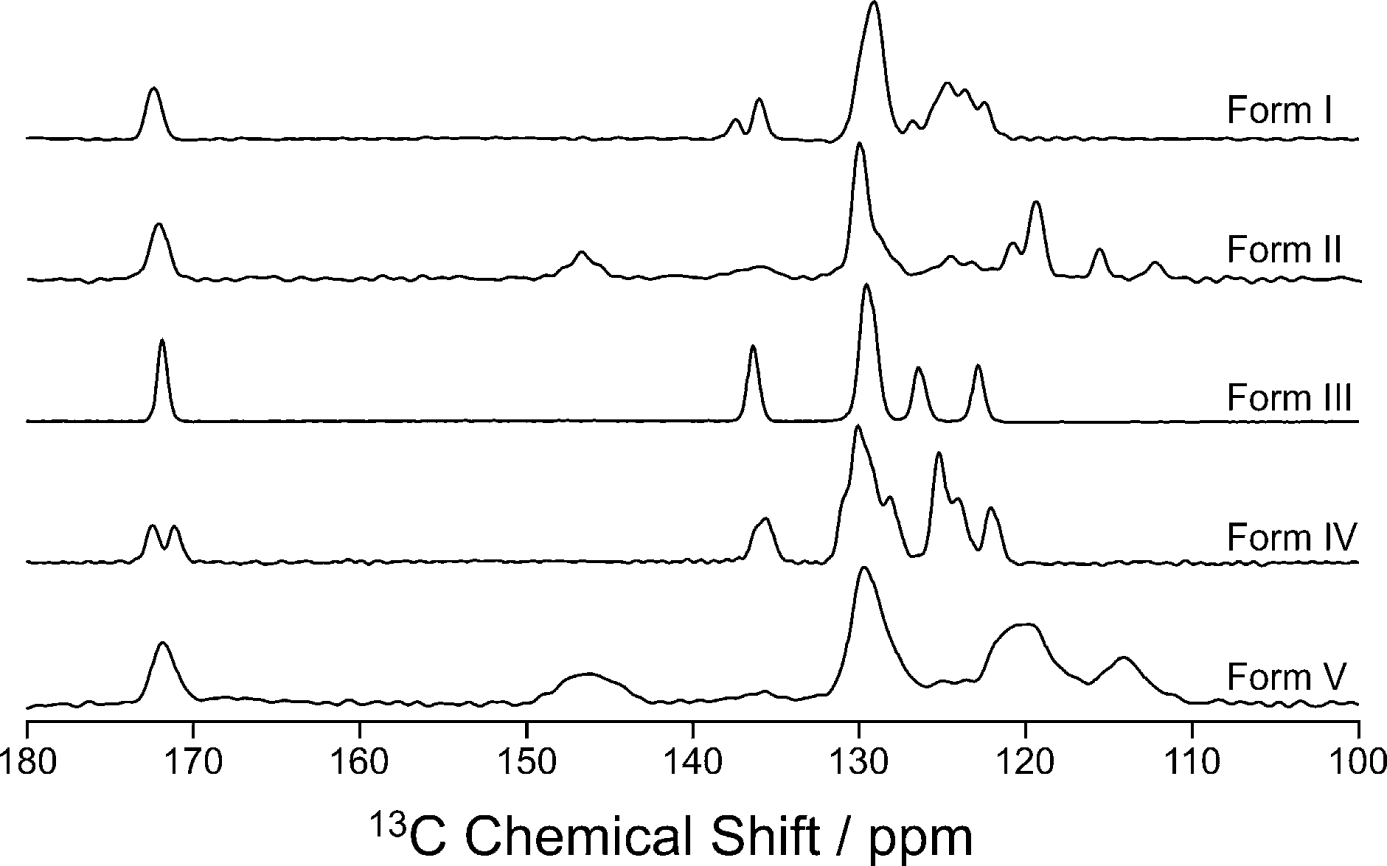
Solid-state ^1^H→^13^C CPMAS NMR spectra for powder samples of the five polymorphs of *m*-ABA.

The CLASSIC NMR strategy is applied here in ^13^C NMR studies of crystallization of *m*-ABA from dimethyl sulfoxide (DMSO),^8^ using a Bruker AVANCE III spectrometer at the UK 850 MHz Solid-State NMR Facility (^13^C, 213.82 MHz; 4 mm HXY probe; zirconia rotor suitable for spinning liquid samples; MAS frequency 12 kHz). The solution was initially held at 120 °C for 1 h to ensure complete dissolution, then cooled to 33 °C over ca. 15 mins. The CLASSIC NMR strategy was then applied over 15 h. The time to record each spectrum was 38.4 mins for ^13^C CPMAS and 6.4 mins for ^13^C direct-excitation. Thus, the effective time resolution for the CLASSIC NMR study was 44.8 mins. More details are given in the Supporting Information.

The evolution of the solid-state ^13^C NMR spectrum is shown in Figure 3. The first signal emerged ca. 2 h after commencing the experiment, signifying the start of crystallization. From the ^13^C chemical shifts, the solid phase is assigned as Form I of *m*-ABA. The intensity then increased monotonically with time (Figure 4), indicating an increase in the amount of solid, levelling off at ca. 8 h. No change in ^13^C chemical shifts was observed with time, indicating that only Form I was present during the crystallization process. While no polymorphic transformation occurs in this case, we emphasize that CLASSIC NMR would be able to identify any changes that occur in the identity of the solid phase, as demonstrated in previous in-situ solid-state NMR studies of crystallization3a,b (i.e., involving only the solid-state component of the CLASSIC NMR strategy). Indeed, in-situ solid-state ^13^C NMR has shown that, in crystallization of *m*-ABA from methanol, Form I is produced in the early stages before a polymorphic transformation occurs to give Form III (the final crystallization product) in the later stages of the crystallization process (see Supporting Information for more details).

**Figure 3:**
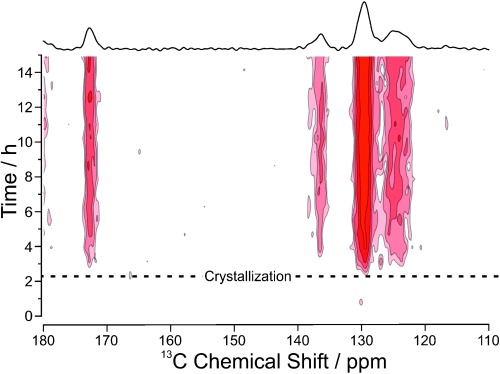
Solid-state (^1^H→^13^C CPMAS) component of the CLASSIC ^13^C NMR data. The sum of all spectra (shown at top) is identified as Form I of *m*-ABA, with no evidence that any other polymorph was present during the process.

**Figure 4:**
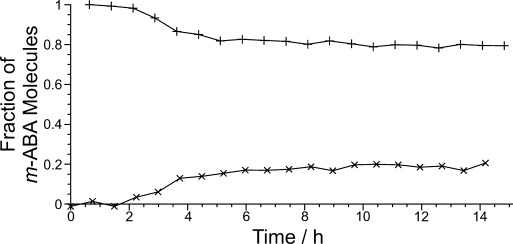
Fraction of *m*-ABA molecules in the liquid (+) and solid (×) phases as a function of time, established from the integrals of the liquid-state and solid-state components of the CLASSIC NMR data respectively.

The liquid-state ^13^C NMR spectrum has sharp peaks for each of the seven ^13^C environments in *m*-ABA, with only J-coupling to directly bonded ^1^H nuclei resolved. The total integral of the liquid-state ^13^C NMR spectrum (Figure 4), which allows the concentration of *m*-ABA in solution to be monitored, was constant until the time (ca. 2 h) at which the first signal was observed in the solid-state ^13^C NMR spectrum.^9^ The total integral then decreased with time, before reaching a constant value at ca. 8 h.

Significantly, the results from both the solid-state and liquid-state components of the CLASSIC NMR data are in good agreement that crystallization commenced ca. 2 h after the start of the experiment. Crystal growth then continued for the next ca. 6 h. Thereafter, the total amounts of *m*-ABA in the liquid and solid phases showed no further evolution with time suggesting that, by this stage, the system comprised an equilibrium saturated solution.

We now develop a more detailed interpretation of the changes in the liquid-state ^13^C NMR spectrum during crystallization. Figure 5 shows the ^13^C chemical shift *δ_i_*(*t*) for each site (*i*) in *m*-ABA as a function of time relative to the corresponding initial value *δ_i_*^start^. Initially, the system is a supersaturated solution^10^ (concentration ca. 1.4 times the solubility of Form III at 33 °C). After crystallization begins, the supersaturation decreases with time. By the end of the crystallization process, the system is an equilibrium saturated solution [chemical shifts denoted *δ_i_*^eq^(DMSO)]. To rationalize the changes in the solution state as crystallization proceeds, we consider values of Δ*δ_i_*^classic^=*δ_i_*^start^−*δ_i_*^eq^(DMSO), representing the difference in each chemical shift between the initial maximally supersaturated solution and the final equilibrium saturated solution. Values of Δ*δ_i_*^classic^ determined from the data in Figure 5 are given in Table 1.

**Figure 5:**
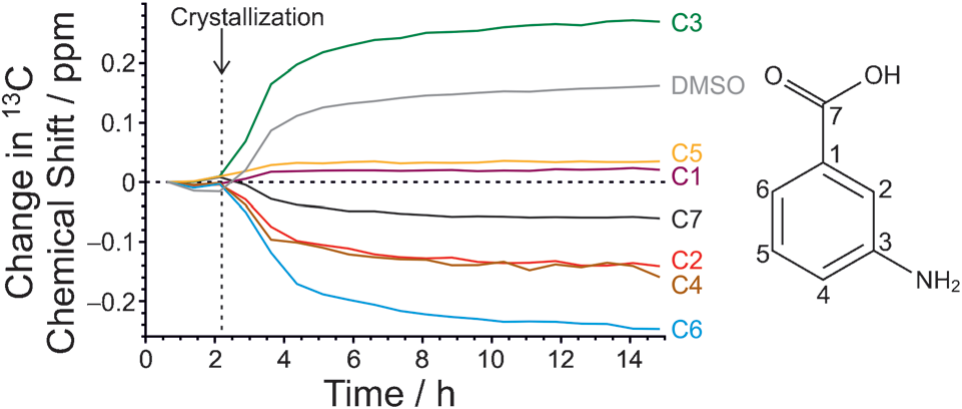
Evolution of ^13^C chemical shifts in the liquid-state (^13^C direct-excitation) component of the CLASSIC NMR data. The vertical dashed line indicates the time at which crystallization commenced (see also Figure 3).

**Table 1:** Values of Δ*δ_i_*^classic^ measured from the liquid-state component of the CLASSIC ^13^C NMR data for crystallization of *m*-ABA from DMSO and values of Δ*δ_i_*^solvent^ determined from liquid-state ^13^C NMR data

	Δ*δ_i_*^classic^	Δ*δ_i_*^water[a]^	Δ*δ_i_*^methanol[a]^	Δ*δ_i_*^1,4-dioxane[a]^
C3	−0.27	−11.48	−0.27	−0.13
C5	−0.03	0.34	−0.30	−0.32
C1	−0.02	3.47	0.01	−0.34
C7	0.06	3.65	1.07	−1.10
C2	0.14	5.09	0.94	0.06
C4	0.16	4.44	0.84	−0.43
C6	0.25	7.27	1.19	0.39
*r*^2[b]^		0.88	0.71	0.05

[a] For saturated solutions of *m*-ABA in water, methanol, 1,4-dioxane and DMSO. [b] Correlation coefficients (*r*^2^) between values of Δ*δ_i_*^classic^ and values of Δ*δ_i_*^solvent^ for each solvent.

Independently, we have recorded liquid-state ^13^C NMR spectra for saturated solutions of *m*-ABA in water, methanol, 1,4-dioxane and DMSO. For each ^13^C site *i* in *m*-ABA, the *difference* in chemical shift in a given solvent relative to DMSO is: Δ*δ_i_*^solvent^=*δ_i_*^eq^(solvent)−*δ_i_*^eq^(DMSO). Comparison (Table 1) of values of Δ*δ_i_*^solvent^ and values of Δ*δ_i_*^classic^ determined from our CLASSIC NMR study of crystallization of *m*-ABA from DMSO gives a very high correlation coefficient for water, a moderately high correlation coefficient for methanol and a very low correlation coefficient for 1,4-dioxane. Recalling that the definitions of Δ*δ_i_*^classic^ and Δ*δ_i_*^solvent^ have the *same* reference point [i.e., chemical shifts *δ_i_*^eq^(DMSO) for a saturated solution of *m*-ABA in DMSO], the high correlation coefficients for water and methanol suggest that the speciation and intermolecular environment of *m*-ABA molecules in the supersaturated DMSO solution at the start of the CLASSIC NMR experiment (reflected in the values of *δ_i_*^start^ and hence in the values of Δ*δ_i_*^classic^) may bear some resemblance to those of *m*-ABA molecules in saturated solutions in water and methanol [reflected in the values of *δ_i_*^eq^(solvent) and hence in the values of Δ*δ_i_*^solvent^]. Based on the high correlation coefficients observed between values of Δ*δ_i_*^classic^ and values of Δ*δ_i_*^water^ and Δ*δ_i_*^methanol^, we now develop two hypotheses concerning the nature of the supersaturated solution of *m*-ABA in DMSO at the start of the CLASSIC NMR experiment.

First, the key difference (reflected in values of Δ*δ_i_*^water^) between saturated solutions of *m*-ABA in water and DMSO is that the *m*-ABA molecules are predominantly zwitterionic in saturated water solution but non-zwitterionic in saturated DMSO solution. Thus, one hypothesis is that, at sufficiently high supersaturation of *m*-ABA in DMSO, the proportion of zwitterionic *m*-ABA molecules is significantly higher than in an equilibrium saturated solution of *m*-ABA in DMSO.^11^

Second, a key difference (reflected in values of Δ*δ_i_*^methanol^) between saturated solutions of *m*-ABA in methanol and DMSO is the presence of a hydrogen-bond donor in methanol. In saturated methanol solution, *m*-ABA is almost entirely non-zwitterionic and the NH_2_ group is likely to act as an acceptor in O–H⋅⋅⋅N hydrogen bonds. Thus, a second hypothesis is that, in the supersaturated solution of *m*-ABA in DMSO at the start of the crystallization experiment, there is an increase in the proportion of non-zwitterionic *m*-ABA molecules present in aggregates containing intermolecular hydrogen bonds with the NH_2_ group as the acceptor.^12^

Thus, the changes (Δ*δ_i_*^classic^) in ^13^C chemical shifts observed in the crystallization process are consistent with the supersaturated solution of *m*-ABA in DMSO at the start of crystallization having a higher proportion of zwitterionic *m*-ABA molecules and/or a higher proportion of non-zwitterionic *m*-ABA molecules present in hydrogen-bonded aggregates, relative to a saturated solution of *m*-ABA in DMSO. Both scenarios represent an increased degree of protonation of the NH_2_ group, leading to increased positive charge on the N atom, increasing the shielding of C3 and promoting the specific changes in ^13^C chemical shifts observed.

Finally, the evolution of the ^13^C chemical shift for the solvent DMSO with time in the liquid-state component of the CLASSIC NMR data (Figure 5) is a direct consequence of the decrease in the concentration of *m*-ABA molecules and hence a decrease in the extent to which DMSO molecules are engaged as the acceptor in O–H⋅⋅⋅O or N–H⋅⋅⋅O hydrogen bonds with *m*-ABA molecules.

Although the crystal structure of Form I of *m*-ABA has not yet been determined, N(1s) XPS studies indicate that the molecules are zwitterionic;5b thus, the structure is expected to contain O^−^⋅⋅⋅H–N^+^ hydrogen bonds as the NH_3_^+^ and CO_2_^−^ groups are the only hydrogen bond donors and acceptors present. The liquid-state ^13^C NMR data suggest that the supersaturated solution at the start of crystallization from DMSO contains 1) zwitterionic *m*-ABA molecules, which may or may not be present as aggregates containing O^−^⋅⋅⋅H–N^+^ hydrogen bonds (although such aggregates must ultimately be formed at some stage on the crystallization pathway) and/or 2) aggregates of non-zwitterionic *m*-ABA molecules linked by O–H⋅⋅⋅N hydrogen bonds. Clearly, either situation is a plausible precursor to the O^−^⋅⋅⋅H–N^+^ hydrogen bonds that must exist between *m*-ABA zwitterions in the crystal structure of Form I. Although we cannot distinguish whether situation (1) or situation (2) is predominant, our results nevertheless give clear insights into the nature of the speciation and interactions that exist in the supersaturated pre-nucleation solution of *m*-ABA in DMSO prior to crystallization, relative to those in the saturated solution at the end of the crystallization process.^13^

The CLASSIC NMR experiment extends the scope and capability of in-situ monitoring of crystallization processes, as it provides complementary information on the time-evolution of both the solid and liquid phases. In spite of the simplicity of the concept of “interleaving” the measurement of liquid- and solid-state NMR spectra, it is surprising that this strategy has not been exploited more widely in other fields, although a notable case is the SedNMR technique, which has been developed to monitor the kinetics of fibrilization and sedimentation processes in biological systems.^14^ Another recent development to study heterogeneous biomaterials is the CMP^15^ technique; however, in contrast to CLASSIC NMR, the CMP technique requires new and specialized probe design and has not been applied to systems containing free liquid nor to study time-dependent processes. In the study of crystallization processes, the CLASSIC NMR technique reported here is unique as an in-situ NMR strategy for simultaneously mapping the time-evolution of both the liquid and solid phases. We anticipate that the advantages of the CLASSIC NMR strategy will yield significant new insights on a wide range of other crystallization systems in the future.

## Supporting Information

As a service to our authors and readers, this journal provides supporting information supplied by the authors. Such materials are peer reviewed and may be re-organized for online delivery, but are not copy-edited or typeset. Technical support issues arising from supporting information (other than missing files) should be addressed to the authors.

miscellaneous_information
