# Supplementary material for: “CLASSIC NMR”: An In-Situ NMR Strategy for Mapping the Time-Evolution of Crystallization Processes by Combined Liquid-State and Solid-State Measurements
Source: Angew Chem Int Ed Engl. 2014 Jul 7;53(34):8939–43. doi: 10.1002/anie.201404266 (PMC4227553; doi:10.1002/anie.201404266)
Supplement: Supplementary file 1 — miscellaneous_information [file anie0053-8939-SD1.pdf]

Angewandte Chemie
Eine Zeitschrift der Gesellschaft Deutscher Chemiker

Supporting Information

© Wiley-VCH 2014

69451 Weinheim, Germany

“CLASSIC NMR”: An In-Situ NMR Strategy for Mapping the Time-Evolution of Crystallization Processes by Combined Liquid-State and Solid-State Measurements**

*Colan E. Hughes, P. Andrew Williams, and Kenneth D. M. Harris**

anie_201404266_sm_miscellaneous_information.pdf

Laboratory Crystallization Experiments

Before carrying out our *in-situ* NMR studies of crystallization of *m*-ABA from DMSO, extensive tests of the crystallization process (involving dissolution at elevated temperature and inducing crystallization by cooling to ambient temperature) were carried out under normal laboratory conditions. In some cases, crystallization proceeded over a period of several hours to give Form I of *m*-ABA. In other cases, however, the viscous, super-saturated solution obtained on cooling to ambient temperature remained kinetically stable and no crystallization was observed within the timescale (up to several days) of our experiments. In such cases, crystallization could not even be induced by agitation, shock or further cooling (to *ca*. –5 °C).

Details of CLASSIC NMR Experiments

The CLASSIC NMR strategy is applied here in ^{13}C NMR studies of crystallization of *m*-ABA from DMSO, carried out on a Bruker AVANCE III spectrometer at the UK 850 MHz Solid-State NMR Facility at the University of Warwick (^{13}C Larmor frequency, 213.82 MHz; 4 mm HXY probe in double-resonance mode; zirconia rotor sealed with an insert tightened into place by a screw, followed by the drive tip). To prepare the crystallization solution (4.5 M), solid *m*-ABA Form III (24.8 mg) was placed at the bottom of the NMR rotor and DMSO (23.2 mg) was added by pipette at ambient temperature. The rotor was then centrifuged for a few seconds to force the sample to the bottom, before inserting sealing caps to prevent leakage of liquid from the rotor under MAS. The MAS frequency was 12 kHz. The rotor was heated to 120 °C for one hour to ensure complete dissolution. The rotor was then cooled to 33 °C over *ca*. 15 mins, before commencing the CLASSIC NMR measurements. To record the solid-state ^{13}C NMR spectrum, ramped ^{1}H→^{13}C CP[1] was employed (CP contact time, 1.5 ms) with ^{1}H decoupling using SPINAL-64[2] (nutation frequency, 91 kHz). To record the liquid-state ^{13}C NMR spectrum, the direct-excitation ^{13}C NMR pulse sequence was used, with a single 90° ^{13}C pulse and no ^{1}H decoupling. The recycle delay was 9 s for the CP measurements and 3 s for the direct-excitation measurements. From our observations, there is no evidence (e.g., from diminution of signal intensity within the early scans of the acquisition) to suggest that the recycle delay for the direct-excitation experiments is shorter than five times $T_1(^{13}\mathrm{C})$ for any of the ^{13}C environments in the *m*-ABA molecules in solution. However, we note that direct

measurement of the T_1(^{13}C) values for the super-saturated solutions that exist during the *in-situ* study of the crystallization process are not straightforward, given the meta-stable nature of these solutions and the fact that the T_1(^{13}C) values are expected to vary as a function of time during the crystallization process as a consequence of changes in the solution-state concentration and speciation.

The temperature of the sample was estimated from a lead nitrate calibration,[3] with MAS at 12 kHz found to correspond to a temperature increase of 13 °C. This calibration was corroborated by recording ^{1}H NMR spectra for methanol, for which the chemical shift difference between the two ^{1}H resonances is temperature dependent[4] (while this calibration method was exploited previously for solid-state measurements by soaking the methanol into TTMSS,[5] neat methanol was used inside a sealed rotor in the present case).

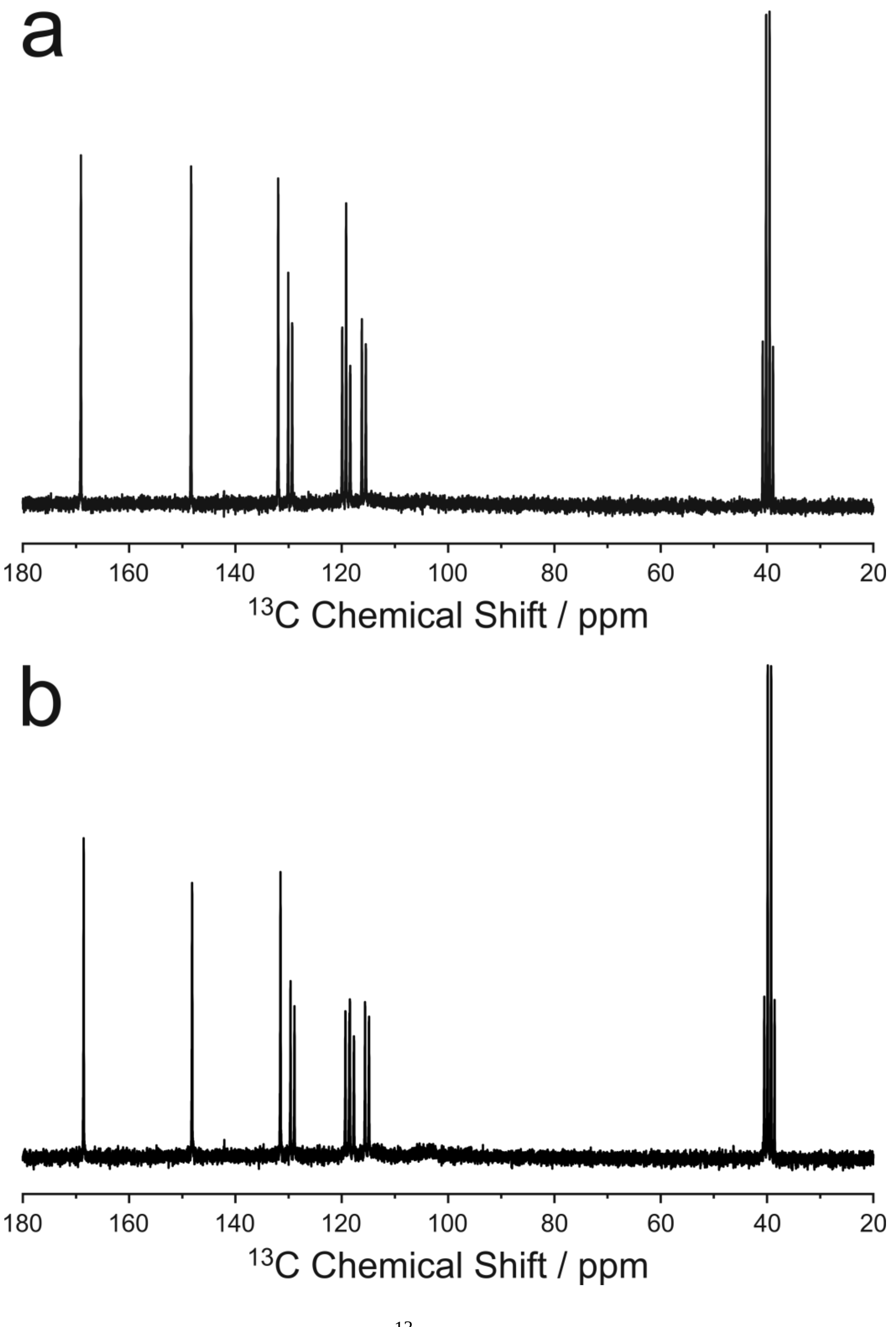

Figure S1 Direct-excitation (liquid-state) ^{13}C NMR spectra recorded in our CLASSIC NMR study of crystallization of *m*-ABA from DMSO. The first liquid-state ^{13}C NMR spectrum is shown in (a) and the last liquid-state ^{13}C NMR spectrum (recorded 14.8 hrs after commencing the experiment) is shown in (b). From comparison of (a) and (b), it is clear that the ^{13}C NMR spectrum shown in (b) does not contain any contribution from the solid phase of *m*-ABA that was present at this stage of the crystallization experiment.

Analysis of CLASSIC NMR Data, Including Methodology for Fitting the Chemical Shift Data

In the ^{13}C NMR data recorded in the CLASSIC NMR experiment, there is a general drift of peaks over time. The magnitude of the drift decreases approximately exponentially with time, converging on a final peak position at lower ppm than the initial peak position. In addition, there is a secondary effect, starting after approximately 2 hrs, which increases or decreases the drift away from a single exponential. The overall drift is attributed to the fact that the magnetic shims take several hours to cool, thus altering the field generated over this period of time. The secondary effect is assigned to changes in concentration due to crystallization, as it begins at the same time that the first peaks are observed in the solid-state (^{13}C CPMAS) spectra. The contribution due to the magnetic shims is removed using a fit to a bi-exponential model.

To remove the effect of the field drift due to slow cooling of the magnetic shims during the crystallization experiment, the changes in isotropic peak positions were fitted to a bi-exponential model with the function:

$$\Delta\delta_i(t) = xe^{-t/\tau_1} \qquad t < t_c$$

$$\Delta\delta_i(t) = xe^{-t/\tau_1} + y_i e^{-(t-t_c)/\tau_{2,i}} \qquad t > t_c$$

where the term xe^{-t/τ_1} models the field drift due to the shims and the term $y_i e^{-(t-t_c)/\tau_{2,i}}$ models the concentration effect. The values of x, τ_1 and t_c were the same for all eight peaks but the values of y_i and $\tau_{2,i}$ were allowed to vary independently for each peak position (denoted i). The term t_c is the time at which concentration effects begin. The result of this fit (with 19 variables) is an exponential curve given by xe^{-t/τ_1} (x = –0.46 ppm, τ_1 = 2.20 hr) which may be subtracted from the plots shown in Figure S2 to reveal the nature of the concentration effects, as shown in Figure S3 (Figure 5 in the paper).

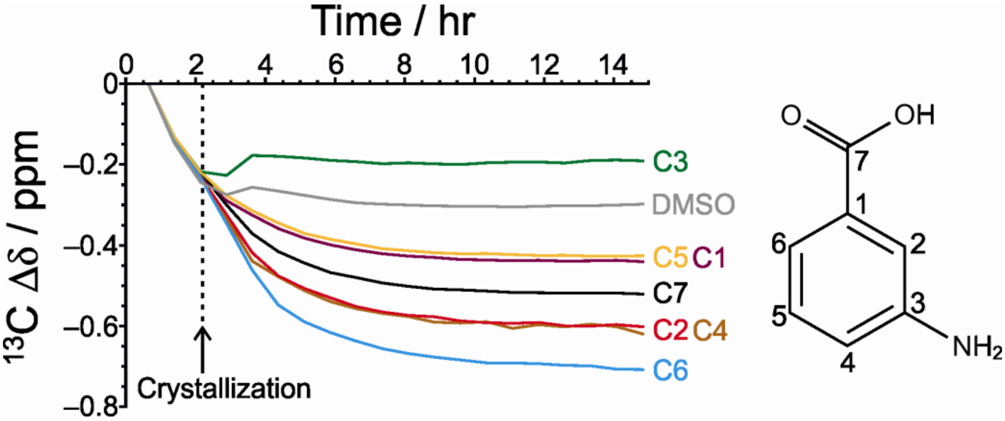

Figure S2 Change in isotropic ^{13}C chemical shifts in the ^{13}C direct-excitation NMR spectra recorded as a function of time during crystallization of *m*-ABA from DMSO, relative to the ^{13}C chemical shift in the first spectrum recorded at t = 38.4 min. The dashed line shows the time (*ca*. 2 hrs) at which crystallization begins, calculated by fitting the curves described above.

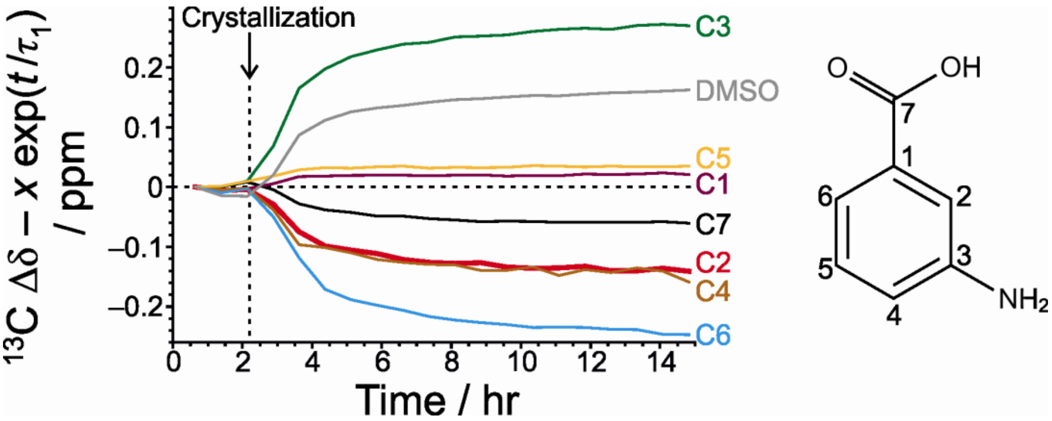

Figure S3 The corrected evolution of ^{13}C chemical shifts in the liquid-state (^{13}C direct-excitation) component of the CLASSIC ^{13}C NMR data, recorded *in situ* as a function of time during crystallization of *m*-ABA from DMSO.

To calculate the fraction of molecules in the liquid-state and the fraction of molecules in the solid-state (Figure 4 of the paper), the following method was used. To determine the fraction of molecules in the liquid state, the integrals of the liquid-state ^{13}C NMR spectra were normalized relative to the integral of the first spectrum recorded (at this stage of the experiment, it is known that all *m*-ABA molecules are in the liquid state). To determine the fraction of molecules in the solid state, the integrals of the solid-state NMR spectra were normalized by setting the fraction (F_S^{eq}) of *m*-ABA molecules in the solid state at the end of the experiment as $F_S^{eq} = 1 - F_L^{eq}$, where F_L^{eq} is the fraction of *m*-ABA molecules in the liquid state at the end of the experiment.

Solid-State ^{13}C NMR Studies of the Five Polymorphs of *m*-ABA

To characterize the solid-state ^{13}C NMR properties of the five known polymorphs of *m*-ABA prior to our CLASSIC NMR studies, each polymorph was prepared using the procedures reported previously.[6] The high-resolution solid-state ^{13}C NMR spectrum was recorded for a powder sample of each polymorph using a Chemagnetics Infinity Plus spectrometer [^{1}H Larmor frequency, 300.2 MHz (75.48 MHz for ^{13}C); 4 mm zirconia rotor; MAS frequency 12 kHz; ramped ^{1}H→^{13}C CP[1] with contact time, 2 ms; TPPM ^{1}H decoupling[7]]. Different recycle delays were used for each polymorph and were at least five times T_1(^{1}H). The value of T_1(^{1}H) for each polymorph was determined by applying inversion recovery on the ^{1}H channel followed by ^{1}H→^{13}C CP and acquisition of the ^{13}C NMR spectrum.

Liquid-State ^{13}C NMR Studies of Saturated Solutions of *m*-ABA

To characterize the liquid-state ^{13}C NMR properties of *m*-ABA in different solvents, solution-state ^{13}C NMR spectra were recorded at 33 °C for saturated solutions prepared by dissolution of Form III of *m*-ABA in water (6.5 mg/g), methanol (71.0 mg/g), DMSO (745.25 mg/g) and 1,4-dioxane (62.6 mg/g). In all cases, the data were recorded on a 500 MHz Bruker solution-state NMR spectrometer. The solution was contained in a 5 mm glass tube and was deuterium locked using D_2O in a sealed glass insert.

In-Situ Solid-State ^{13}C NMR Study of Crystallization of *m*-ABA from Methanol

Our *in-situ* solid-state ^{13}C NMR study of crystallization of *m*-ABA from methanol was carried out using a Bruker AVANCE III spectrometer at the UK 850 MHz Facility at the University of Warwick (^{13}C Larmor frequency, 213.82 MHz; 4 mm HXY probe in double-resonance mode). A sample of *m*-ABA with natural isotopic abundances was used. To prepare the crystallization solution, solid *m*-ABA (Form III, 5.5 mg) was placed at the bottom of a zirconia NMR rotor and methanol (40.8 mg) was added by pipette at ambient temperature. The rotor was then centrifuged for a few seconds to force the sample to the bottom, before inserting sealing caps to prevent leakage of liquid from the rotor under MAS. For the *in-situ* solid-state ^{13}C NMR study of the crystallization process (Figure S4), the MAS frequency was 12 kHz. The rotor was heated to 85 °C for one hour to

ensure complete dissolution. The rotor was then cooled to 33 °C and high-resolution solid-state ^{13}C NMR spectra were acquired repeatedly, using ramped $^{1}H \rightarrow ^{13}C$ CP (CP contact time, 1.5 ms) with ^{1}H decoupling using SPINAL-64 (nutation frequency, 91 kHz). Each spectrum involved the acquisition of 256 scans, with a recycle delay of 9 s. The total time to record each spectrum was 38.4 mins, representing the time resolution of the *in-situ* study.

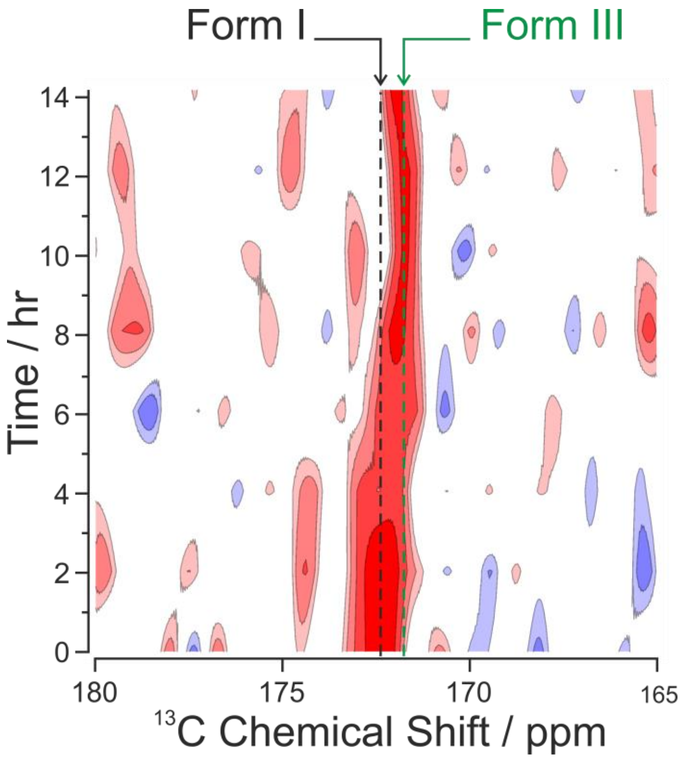

Figure S4 *In-situ* solid-state ^{13}C NMR spectra (showing only the region for the carboxylate/carboxylic acid group) recorded as a function of time during crystallization of *m*-ABA from methanol. The initial crystallization product is identified as Form I (black dashed line), which subsequently undergoes a polymorphic transformation to produce Form III (green dashed line). After *ca*. 9 hrs, the crystallization product is a monophasic sample of Form III.

References

[1] G. Metz, X. L. Wu, S. O. Smith, *J. Magn. Reson. A* **1994**, *110*, 219.

[2] B. M. Fung, A. K. Khitrin, K. Ermolaev, *J. Magn. Reson.* **2000**, *142*, 97.

[3] a) A. Bielecki, D. P. Burum, *J. Magn. Reson. A* **1995**, *116*, 215; b) T. F. Kemp, G. Balakrishnan, K. J. Pike, M. E. Smith, R. Dupree, *J. Magn. Reson.* **2010**, *204*, 169.

[4] a) A. L. Van Geet, *Anal. Chem.* **1968**, *40*, 2227; b) A. L. Van Geet, *Anal. Chem.* **1970**, *42*, 679.

[5] A. E. Aliev, K. D. M. Harris, *Magn. Reson. Chem.* **1994**, *32*, 366.

[6] P. A. Williams, C. E. Hughes, G. K. Lim, B. M. Kariuki, K. D. M. Harris, *Cryst. Growth Des.* **2012**, *12*, 3104.

[7] A. E. Bennett, C. M. Rienstra, M. Auger, K. V. Lakshmi, R. G. Griffin, *J. Chem. Phys.* **1995**, *103*, 6951.
